# Tuberculosis in UK cities: workload and effectiveness of tuberculosis control programmes

**DOI:** 10.1186/1471-2458-11-896

**Published:** 2011-11-28

**Authors:** Graham H Bothamley, Michelle E Kruijshaar, Heinke Kunst, Gerrit Woltmann, Mark Cotton, Dinesh Saralaya, Mark A Woodhead, John P Watson, Ann LN Chapman

**Affiliations:** 1 Homerton University Hospital, London, UK; 2 Health Protection Agency - Health Protection Services Colindale, London, UK; 3 Birmingham Heartlands Hospital, Birmingham, UK; 4 Glenfield Hospital, Leicester, UK; 5 Glasgow Royal Infirmary, Glasgow, UK; 6 Bradford Royal Infirmary, Bradford BD9 6RJ, UK; 7 Manchester Royal Infirmary, Manchester, UK; 8 St James's University Hospital, Leeds, UK; 9 Royal Hallamshire Hospital, Sheffield, UK

## Abstract

**Background:**

Tuberculosis (TB) has increased within the UK and, in response, targets for TB control have been set and interventions recommended. The question was whether these had been implemented and, if so, had they been effective in reducing TB cases.

**Methods:**

Epidemiological data were obtained from enhanced surveillance and clinics. Primary care trusts or TB clinics with an average of > 100 TB cases per year were identified and provided reflections on the reasons for any change in their local incidence, which was compared to an audit against the national TB plan.

**Results:**

Access to data for planning varied (0-22 months). Sputum smear status was usually well recorded within the clinics. All cities had TB networks, a key worker for each case, free treatment and arrangements to treat HIV co-infection. Achievement of targets in the national plan correlated well with change in workload figures for the commissioning organizations (Spearman's rank correlation R = 0.8, P < 0.01) but not with clinic numbers. Four cities had not achieved the target of one nurse per 40 notifications (Birmingham, Bradford, Manchester and Sheffield). Compared to other cities, their loss to follow-up during treatment was usually > 6% (χ^2 ^= 4.2, P < 0.05), there was less TB detected by screening and less outreach. Manchester was most poorly resourced and showed the highest rate of increase of TB. Direct referral from radiology, sputum from primary care and outreach workers were cited as important in TB control.

**Conclusion:**

TB control programmes depend on adequate numbers of specialist TB nurses for early detection and case-holding.

Please see related article: http://www.biomedcentral.com/1741-7015/9/127

## Background

The process of improving services requires planning, followed by identification and supply of the required resources, implementation and then monitoring and evaluation [1]. At the national level, surveys of tuberculosis (TB) in England and Wales have occurred every five years from 1978 to 1998 [2,3], and thereafter by annual reports from the Health Protection Agency (HPA) [4]. From these data, a plan to improve TB control was published in 2004 [5]. The 2010 HPA report reviewed progress against the plan. Improvements in generic processes included the use of web-based enhanced surveillance data collection, guidelines on treatment and management of tuberculosis [6], agreement on service provision [7], availability of information in different languages from the Department of Health publications and charities, improvements in prison health services and standards for mycobacterial reference laboratories [8], including DNA fingerprinting of all UK strains from April 2010. Guidance on the management of complex cases and clarification of the role of mass x-ray screening is currently being undertaken.

The aim of this study was to examine how the national plan has been translated into a control program for the larger cities within the United Kingdom (UK). In London, Birmingham and Manchester several TB services and hospitals combine to execute TB control. In other cities, a single TB service is responsible for delivering the TB control plan. However, in each city, a combination of public health specialists, physicians, TB nurses and local authorities are required to act jointly to enable effective TB control. This paper compares cities with a continuing increase in numbers of TB cases to those where numbers are stable or falling in order to identify gaps in services. The time course of TB control requires data over a period of at least 10 years as both early disease and late reactivation may occur following infection [9]. A subsidiary aim was to identify reasons which could account for differences in the change in TB notifications in these large cities.

## Methods

### Cities

The 10 most populous urban areas in the United Kingdom are described with data from the 2001 census. The areas, as defined by their primary care trust (PCT) and associated with an individual TB service, with an average of ~100 cases per year were then identified and those which were in the most populous cities were invited to take part in the survey.

### Data

Notification of tuberculosis is a legal requirement in the UK. Data from the national enhanced TB surveillance (ETS) are collated by the HPA and reports prepared annually http://www.hpa.org.uk. Collective data without personal indicators (anonymized) from individual clinics are available as part of the hospital's activity analysis and for planning purposes by the commissioners of health services; this manuscript would count as a response to a request under the Freedom of Information Act for data regarding TB services in individual clinics. Each clinic was asked to provide the number of TB notifications and number of sputum smear-positive cases over a period of at least 10 years, ethnicity of population, HIV co- infection (where available), strain typing data and treatment outcome. Data of sufficient duration to identify the inflexion point at which the expected exponential decline in TB incidence was reversed and/or where the increase in TB cases began to decline were requested. Each physician was asked to interpret the trends, including any additional factors they thought were important locally.

Rates were obtained using available population figures (ETS). At the operational level, a TB team has to manage the identified patients and estimate future requirements for their service from their characteristics. Hence, the workload for a TB team has been defined by the number of patients diagnosed and treated for TB. In the UK, the TB nurse has two main responsibilities - contact tracing and supervision of treatment. Contact tracing is usually performed in outpatients to ensure access to chest x-rays, blood tests and tuberculin skin testing and proximity to a doctor, who can diagnose active or latent TB and initiate appropriate treatment. Each TB patient should have a key worker, usually a TB nurse, responsible for ensuring adherence to treatment. This may include home visits, monitoring or providing directly observed therapy at whatever location is feasible. The doctor or the TB nurse is responsible for the notification of a case of TB to public health and this may be performed by an administrator. Public health coordinates contact tracing in non-household contacts, such as at work or in a school outbreak. Screening high risk groups, such as immigrants, and outreach work may also be part of the TB nurse's role, but is a lower priority. The TB team may also include outreach or community health workers with responsibility for particular cultural groups, especially those whose first language is not English, or "hard to reach" individuals such as the homeless and those with an alcohol or drug problem. In these circumstances, the outreach or community health worker may then be the key worker or accountable case manager. These functions have been included as items from the TB Action Plan and information regarding these was obtained from each TB service.

### Analysis

Data were plotted against time. In order to determine the annual percentage changes, a trend line was calculated in Powerpoint using an exponential gradient on the grounds that changes in an infectious disease should be geometric rather than linear when the reproduction rate is > 1 (reproduction rates for TB have been estimated at 5.16 ± 2.82 [9]). A chi-squared test was used to compare achievement of adequate TB nursing with loss to follow-up. Spearman's rank correlation was used to assess whether any individual variable could relate to the trend in TB numbers, smear-positive cases or TB cases with white ethnicity.

## Results

### UK cities and tuberculosis

Major conurbations were identified from the 2001 census (Table 1).

**Table 1:** Major conurbations in the UK and smaller cities with high TB burden

City	**Population**^**1**^	Population density (person/ **hectare)**^**1**^	Actual population of conurbation covered by TB clinics in 2009	**No of TB cases in 2009**^**2**^	Incidence per 100, 000
London	8, 278, 251	51.0	7, 747, 748	3, 440	44.4

Birmingham	2, 284, 093	38.1	2, 284, 093	509	22.3

Manchester	2, 240, 230	40.3	419, 628	248 (196)	59.1 (40.5)

West Yorkshire	1, 499, 465	40.5	Leeds: 762, 461 Bradford: 467, 363	124 179	15.7 38.3

Glasgow	1, 168, 270	40.5	866, 379	213	24.2

Newcastle	879, 996	41.7	268, 751	43	16.0

Liverpool	816, 216	43.9	433, 333	52	12.0

Nottingham	666, 358	42.0	909, 836	86	9.5

Sheffield	640, 720	39.5	530, 000	106 (80)	20.0 (14.6)

Edinburgh	448, 624	37.7	452, 514	81	17.9

Leicester	441, 213	43.4	304, 598	212	69.6

Coventry	300, 848	31.1	312, 925	92	29.4

^1^Office of National Statistics 2001 census.

^2^Numbers supplied by lead TB physician for the city where participants or from ETS (enhanced TB surveillance): discrepancies in figures were noted for Manchester and Sheffield

The lead TB physician for the city or a representative for London and Birmingham were approached and, where numbers of cases were ~100 per year, asked to provide more detailed data. Within London, 17 and, outside London, 9 Primary Care Trusts (PCTs) fulfilled the criteria. Coventry fulfilled the criteria and had a microbiologist interested in TB, but the single-handed respiratory physician had interests other than TB and there was no infectious diseases service on their web-site. The physician for Sheffield had data suggesting their clinic fulfilled the selection criteria, but these differed to those from ETS; data from both sources is therefore represented (Table 2).

**Table 2:** Number of cases and rates of tuberculosis in selected areas (ETS except where indicated)

Area	2000	2001	2002	2003	2004	2005	2006	2007	2008	2009
**Numbers of TB cases**										

Birmingham East and North^1^	117	92	93	97	123	109	112	102	131	140

Bradford and Airedale	138	147	117	142	110	161	186	172	171	210

Heart of Birmingham^1^	175	171	178	182	213	220	244	219	245	259

Leeds	69	93	101	79	114	106	147	103	152	124

Leicester	148	265	194	200	162	263	227	223	201	212

Central Manchester	114	134	140	118	156	147	161	175	167	196

Sandwell^1^	79	85	86	88	89	119	114	112	110	102

Sheffield (HPA)	64	84	58	91	92	86	102	131	75	80

Sheffield (clinician)	81	87	84	105	108	111	118	142	111	

Greater Glasgow	173	161	199	182	208	208	164	172	201	

London Region	2640	2632	2987	3056	3114	3447	3324	3229	3386	3440

**Rate of tuberculosis by year**										

Birmingham East and North^1^		24.8	23.8	24.7	31.2	27.4	28.3	25.4	32.4	34.4

Bradford and Airedale		31.2	24.7	29.8	22.8	33.0	37.7	34.6	34.1	41.4

Heart of Birmingham^1^		56.5	67.7	68.8	80.0	81.6	90.0	80.6	89.1	92.3

Leeds		13.0	14.0	10.9	15.6	14.3	19.6	13.5	19.7	15.7

Leicester		93.7	68.7	70.9	57.2	91.9	78.4	76.2	68.2	69.6

Central Manchester		31.7	32.9	27.4	35.8	33.1	35.6	38.2	36.0	40.5

Sandwell^1^		29.9	30.2	30.8	31.1	41.5	39.6	39.0	38.0	35.1

Sheffield		16.4	11.3	17.7	17.8	16.5	19.4	24.7	14.0	14.6

Greater Glasgow										

London Region	36.5	35.9	40.6	41.5	42.1	46.2	44.2	42.7	44.4	44.4

^1 ^In this study, Birmingham comprised these 3 Primary Care Trusts. Data supplied by Health Protection Agency except where indicated.

Obtaining data across a city was more difficult than expected. The time between a request for data and their receipt for this manuscript from the physician ranged from almost immediate to 22 months. In Glasgow, alteration in boundaries in 2006 had affected the ability to obtain data for a consistent area.

**Figure 1:**
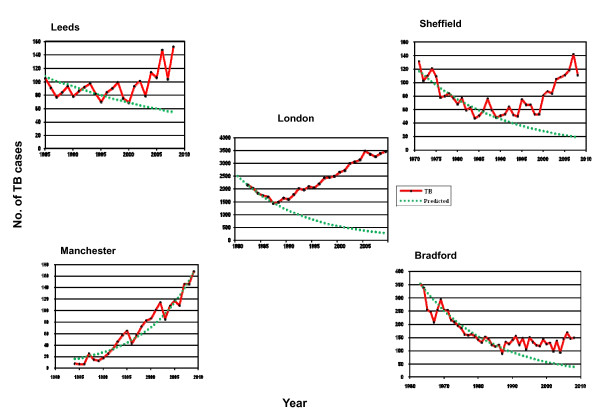
**Comparison of trends and numbers of tuberculosis in UK cities**. Data from Sheffield was supplied by the clinician and varied compared to that available to the enhanced surveillance database.

Physicians provided long term data and in five the duration was sufficient to see when the numbers of TB cases reversed its downward trend (Figure 1). Numbers were steady for Glasgow from 1990, biphasic in Leicester from 2000 and showed an exponential increase in Birmingham from 1990 (data not shown). The rate of change over the decade, the year when the trend reversed and the rate from that point of inflexion were calculated for primary care trusts and their corresponding TB control team in England (Table 3).

**Table 3:** Changes in clinic workload compared to figures for PCT

TB service	TB seen as % local PCT (10 yr average)	Change of TB numbers seen in clinic (% p.a.)		Change in TB by PCT (10 year average, % p.a.)	
		
		Since point of inflexion (year)	10 year average	Numbers	Rates
Birmingham (Heartlands)	94.1	5.4 (1995)	4.3	4.2	5.5

Bradford	88.7	0.6 (1985)	3.7	4.9	8.9

Leeds	100.0	7.6 (1999)	6.7	6.7	4.2

Leicester	129.6	-7.5 (2005)	0.4	4.9	-7.2

London	NA	4.2 (1988)	4.3	4.3	2.1

Manchester (Central)	77.7	12.4 (1985)	6.7	5.2	3.2

Sheffield	133.5	5.4 (1990)	6.0	3.4	1.6

**Figure 2:**
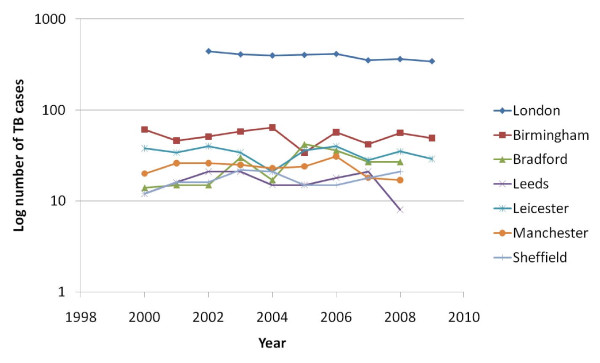
**Numbers of TB cases in white ethnic group by year**.

Data for Greater Glasgow were not available due to changes in boundaries. The rate of change in TB numbers since the point of inflexion was highest for Manchester, Leeds and Sheffield, but lowest for Leicester and the ranking did not change substantially by using the 10 year average for the clinic. PCT data for TB numbers showed a similar trend for the highest three cities, although suggested an increase in cases for Leicester. However, the 10 year rate of change in incidence per 100, 000 was greater in the clinic than in the general population for Leeds, Manchester and Sheffield, very similar for London, Birmingham and Leicester and much lower in the clinic compared to general population in Bradford. Looking at the white ethnic group, Bradford recorded an increase of 8.6% per year, Sheffield an increase of 3.5% until 2008, whilst all the other cities showed a decline, with London showing the greatest rate of decline of 3.3% per year (Figure 2).

Smear-positive pulmonary TB is the main source of infection. Figures available to public health (HPA) often did not contain sputum smear status, probably because data entry to the electronic registers occurred mostly at diagnosis (Table 4).

**Table 4:** Pulmonary cases for which smear results are available and that are sputum smear positive 2000-2009 (Enhanced Tuberculosis Surveillance)

PCT	Pulmonary cases with known sputum smear result (%)				Pulmonary cases with positive sputum smear (%)			
	
	Overall	3-year average			Overall	3-year average		
	
	2000-2009	2001-2003	2004-2006	2007-2009	2000-2009	2001-2003	2004-2006	2007-2009
Birmingham East and North	42.8	46.1	46.1	34.4	91.5	100.0	79.8	95.5

Heart of Birmingham Teaching	38.4	39.7	45.5	31.3	89.3	100.0	75.9	95.1

Bradford and Airedale Teaching	49.9	52.3	55.0	50.2	45.3	59.8	34.6	47.7

Leeds	58.9	60.1	57.1	58.0	42.4	50.0	39.8	40.5

Leicester City	65.0	72.5	62.5	59.5	50.6	51.1	54.9	44.8

Central Manchester	59.9	64.9	69.3	51.3	62.5	58.0	66.0	66.7

Sandwell	65.8	71.9	67.5	61.4	57.5	60.9	51.8	54.6

**Region**								
London	66.3	55.3	69.2	74.1	53.3	57.1	54.6	49.0

Data for Sheffield were not available from ETS.

**Figure 3:**
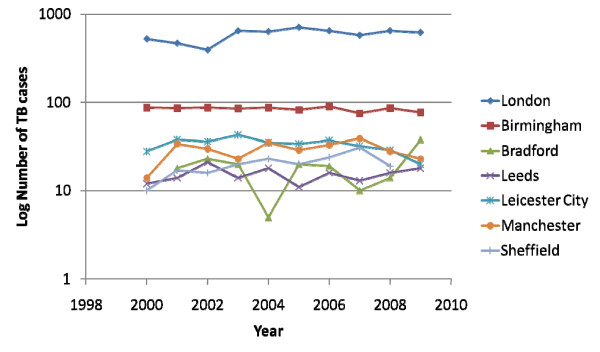
**Smear-positive tuberculosis: numbers and trends**.

However, access to these figures was available to participating clinicians and showed that the numbers of this form of TB have been stable except for an increase in Bradford over the last 5 years and Sheffield with a smaller increase over the last decade (Figure 3).

### Identifying needs

There were gaps in data collection. Sputum smear status would not have been generally well documented in data accessible by the commissioning teams (PCTs), as noted above. In order to address different language groups and cultures with a higher risk of TB, country of origin was found to be more helpful than ethnicity for strategic planning. The general use of the term "Black-African" was especially unhelpful in distinguishing groups such as Somalis from those arriving from other areas of conflict such as the Congo and wealthier West Africans. For instance, in Sheffield, which has one of the oldest and largest established Somali communities in the UK, 25% of all TB between 1999 and 2005 occurred in patients born in Somalia, but in 2009 this proportion had dropped to 10% and those from Eritrea, Ethiopia and Zimbabwe who were 2% of the total caseload in 1999 contributed 23% in 2009. HIV status is not recorded on e-surveillance but is known to clinicians.

Adherence to national guidelines is one way to identify the need for resources. Items from the national TB plan relevant to clinical services were reviewed (Table 5).

**Table 5:** Audit of TB services against items from the national TB Action Plan

Criterion	Birmingham	Bradford	Glasgow	Leeds	Leicester	London	Manchester	Sheffield
1. Have you had a formal peer review against the NICE guidelines?	No	No	No - Scottish guidelines still awaiting agreement	Internal review	No - regular audits and epidemiological review	Formal reviews are carried out in NE and NC London	Annual audit against NICE guidelines	Yes

2. Do you have GP training days with a focus on TB in your area?	Yes	Annual	No	No	No training days solely for TB but regular presentations to GPs as part of other meetings	GP training days are not widely held	No	Yes

3. What did you do for the last World TB Day	Stands at supermarkets, mosques, community centres, four hospitals. Information leaflets about TB was sent to all Birmingham GP's	Nothing	Posters and information leaflets around health centres and hospital	Nothing	Public awareness campaign with representation in the community and specific event organised for primary care with information pack on a CD.	Most TB services carried out public awareness programs	Insufficient staff to do anything	Article in GP communication magazine; TB nurses involved in nurse education, annual evidence reviews and in the past had a stall in main shopping mall.

4. Having identified high risk groups in your area, what sort of educational outreach have you been able to do in the last 2-3 years?	Seminars in nursing and care homes, training of community nurses about TB, educational meeting in certain ethnic communities	Language barriers have prevented outreach work	High risk groups identified (alcohol problems), but no educational outreach	Yes. First in 2011	Some work with community development workers around World TB Day and teaching sessions to community groups. Targeted representation at health fairs	TB Find and Treat has been actively involved in the homeless and intravenous drug users.	Just starting outreach with help of charity, TB Alert.	TB nurses have targeted practices with most TB cases; Somali community addressed by ex-patients and community leaders.

5. Does your local medical school give teaching on TB (do you do it)?	Yes	Teaching given in 3^rd ^and 5^th ^years	Yes - I give 1 lecture a year	TB is in the curriculum and all students that attend St James University Hospital receive TB teaching	Yes. 2^nd ^year lectures and tutorial on TB microbiology and clinical TB. I give a lecture annually.	All five medical schools provide teaching on TB.	Yes - I do it.	Yes - I do it.

6. How often does your TB Network meet and what is its composition?	3-monthly: physicians, paediatricians, TB nurses microbiologists, public health, pharmacists and commissioners	3-monthly Physicians, TB nurses, GP.	6 monthly. Public health (2 CIECs), paediatrician, ID physician, TB nurses, respiratory physicians from each hospital, microbiologist.	a) weekly MDT with TB physician, ID physician, CCDC, TB nurses, microbiologist, PCR technician and pharmacist; b) Leeds TB group quarterly with other hospital teams, primary care managers and co-opted as needed; c) Twice yearly West Yorkshire TB teams	6 weekly meetings with respiratory physician, ID physician, HPA, TB nurses to discuss local epidemiology and difficult cases.	A London TB group has been meeting regularly since 2000. Meetings were initially quarterly, but have increased considerably over the last two years	6-monthly Manchester group. Includes 3 hospitals primary care, public health, TB doctors and nurses, microbiologist, infectious diseases (HIV) doctor.	Regional group meets 1-2× per year. TB forum meets with commissioners 2 × per year. Monthly MDT with infectious diseases, respiratory physicians, TB nurses, laboratory staff, public health and paedicatricians.

7. Do you have a local prison? How many ex- or current prisoners did you treat for TB last year?	Yes 2	No None	Yes. Not recorded.	2 prisons - 1 case last year	2 prisons; 1 case per year.	A TB specialist nurse attached to the prison health service was employed from 2006-2010	Yes. 1-2 patients per year.	Yes (Doncaster); 2 per year

8. Do you have a named key worker (accountable case manager) for each TB patient?	Yes	Yes	Yes	Yes	Yes	Yes	Yes	Yes

9. Are TB drugs free from your clinic?	Yes	Yes	Yes (Scottish national policy)	Yes	Yes	Yes	Yes	Yes

10. How many negative pressure rooms do you have in your hospital?	2 at Trust; 8 for Birmingham	6 at one site	2	4	13 in 2 trust hospitals	Variations in supply exist across London, ranging from 0 to 12 in specialist hospitals	None (available in a different hospital, under a different physician)	17

11. What percentage of TB cases came from screening programmes (contacts, immigrants, HIV_+ _and other)	52/264 (19.7) 52/509 (10.2)	25%	Not available	18/125 (14.4%)	10% from contacts; 75% pulmonary cases identified by radiology based rapid access system	Not recorded across the capital; 11/29 clinics perform new entrant screening^a^	6.9	1-2% per year

12. Do you have a joint TB-HIV clinic?	Yes	Run by infectious diseases physician	No	Just starting	Yes between ID and GUM physicians	All sectors have at least one TB-HIV clinic; only 3 of 29 clinics reported difficulty accessing an HIV service^a^	No, but weekly joint MDT.	Same physician

13. What percentage of your patients had DOT at some point in their treatment last year?	21	0	None	3	5	Access to DOT is variable across London (range 1.7-32% of all patients)^a^	2	5-10%

14. Target of 1 nurse per 40 notifications*	1:80-90 to 2008 1:60-70 from 2008	No specified TB nurses	Not achieved	Achieved	Achieved from 2000	Set 2000 Mostly achieved by 2007; range 1;21 to 1;51 in 2009^a^	Not achieved 2 nurses from 2000	Achieved 2006

15. How many hours are assigned to TB in your job plan?	12	None - TB seen as part of general respiratory clinics	4	8	4	Varies from 0 to 16, but unrelated to TB numbers	2	4 for TB clinic and 12 in total.

*The ratio of notifications per TB nurses was not specified in the national Action Plan, nor in the NICE guidelines.

^a^Data from PHAST report

Abbreviations used: CCDC - consultant in communicable disease control (i.e. public health physician); CIEC - consultant in infection and envornmental control (i.e. public health physician); CD - compact disk; GP - general practitioner (family or primary care doctor); HPA - Health Protection Agency (i.e. public health); MDT - multidisciplinary team; NICE - National Institute for Health and Clinical Excellence; TB - tuberculosis.

All clinics provided TB drugs free of charge and had a named key worker for each person with TB. The majority had a link with HIV physicians, such that patients with HIV co-infection had some form of joint management. All clinics noted that TB education was guaranteed in their local medical schools but postgraduate education of general practitioners was patchy.

All areas outside London have had access to strain typing for a number of years for selected clusters: Sheffield reported the highest proportion of clusters, followed by Birmingham (data not shown). Most strain typing had been performed as dictated by clinical circumstances (drug-resistance or suspicion of enhanced transmission). Routine strain typing has only recently been introduced across the UK.

### Supply of resources

TB networks had been established in all areas by 2009 and included public health in all except Bradford. Until 2011, resources were controlled by the PCTs and services commissioned on the basis of data known to them. There was a correlation between the trend in TB numbers available to PCTs and the number of items successfully completed in the audit (Spearman's rank correlation R^2 ^= 0.64, P < 0.05), whereas correlations with actual trends within the clinic or since the point of inflexion showed less if any correlation. Manchester was significantly under-resourced compared to other cities (Table 5) and showed the greatest rate of increase in clinic numbers (Figure 1; Table 3). There was no consistency in the allocation of time for TB to physicians in their job plans (Table 5).

Four cities (Birmingham, Bradford, Manchester and Sheffield) had not attained the target of 1 nurse per 40 notifications. Clinics with fewer nurses were less likely to use World TB Day as a means of promoting TB awareness or engage in outreach activities. Those cities which had not attained a target were more likely to have > 6% lost to follow-up (χ^2 ^= 4.2, P < 0.05, with Yates' correction; Table 6).

**Table 6:** Outcome and reasons for not completing treatment within 12 months (Enhanced Tuberculosis Surveillance)

Area	2006-2008		2001-2008		
	
	Outcome not reported	Completed within 12 m	Lost	Unknown	Combined*
**PCT**					
Birmingham East and North	4.9	85.1	5.4	5.1	10.5

Heart of Birmingham Teaching	5.5	83.1	6.0	4.7	10.7

Central Manchester	5.8	83.5	6.8	1.8	8.6

Leeds	5.0	80.9	4.4	2.5	6.9

Bradford and Airedale Teaching	5.1	78.1	6.0	0.4	6.4

Sandwell	0.0	76.8	5.4	0.9	6.3

Leicester City	5.7	86.6	4.9	0.8	5.7

Sheffield	11.4	75.8	7.4	3.9	11.3

**Region**					
London	0.02	82.6	5.0	0.7	5.7

*Other reasons for not completing treatment (died, still on treatment, stopped and transferred) are related to an aging population, drug-resistance and planned clinical action respectively. Data for England and Wales supplied by Health Protection Agency; data for Glasgow were not available.

### Implementation

Each clinic had a named key worker, usually the TB nurse, for each patient and was able to supply treatment free of charge. Negative pressure rooms were available to every clinic, but located at a different hospital in Manchester. By 2009, all patients with TB and HIV co-infection had formal relationships with HIV physicians for review of their management.

Screening programmes contributed significantly to the identification of TB (range 2-25% of all TB diagnoses, Table 5). All areas identified contacts of cases of TB. All cities had a new entrant screening programme, but in Sheffield this was limited to asylum seekers. In Leicester since 2004, county-wide reporting of patients with x-ray abnormalities has flagged up 300 suspicious cases per year, 35-40% of whom proved to have active TB.

### Monitoring and evaluation

There was a significant variation in the availability of directly observed therapy (DOT). This reflected the number of TB nurses and also the local populations. Complex cases i.e. homeless, prisoners and those with alcohol, opiate or cocaine addiction were more common in some areas and increased the proportion receiving DOT, especially in London. Outcome reporting has improved significantly over the decade and is almost universal for London and Sandwell (Table 6). Completion rates were < 85% in all areas except Leicester. As noted before, inadequate nurse numbers were associated with a higher rate of loss to follow-up during treatment.

Cities and localities with decreasing TB notifications put forward a range of possible reasons for their improving numbers. Adequate TB nursing numbers, improved treatment completion rates, a good multidisciplinary TB team, improvements in screening for latent TB and re-organisation of TB services across professional/organisational boundaries. Early referral from radiology, sputum samples from primary care and outreach workers to improve case-holding were also cited as important activities in TB control.

A decrease in smear-positive cases has been proposed as a measure of the effectiveness of a TB service on the grounds that early diagnosis should result in fewer with advanced disease. There was no correlation between the change in smear-positive TB numbers and any index of the TB services. There was an inverse association between the change in rate per 100, 000 and the proportion of sputum smear-positive cases except in Leicester, which was a clear outlier (Spearman's rank correlation without Leicester, R^2 ^= 0.69, P < 0.01). A reduction in the white population with TB is thought to indicate effectiveness of TB control, but surprisingly there was a inverse relationship such that clinics with the greatest reduction in TB cases showed higher numbers in the white population (R^2 ^= 0.62, P < 0.05).

## Discussion

Efforts to implement the national TB control programme have been started. Data discrepancies between the clinics and enhanced surveillance need to be addressed, especially the recording of sputum smear status. However, the main obstacle to achieving many of the clinical aims has been the lack of sufficient TB nurses. Commissioners require a projected number of TB cases, calculated from the 10 year rate of change, to ensure adequate resourcing.

### Data collection

Numbers of TB cases continue to increase in the UK, and most TB occurs in large cities (Kruijshaar et al, submitted). This study confirms that this increase varies across the larger cities in the UK. The lack of significant numbers of TB in cities such as Newcastle, Liverpool, Nottingham and Edinburgh may be due to immigration following patterns established by pre-existing communities. Discrepancies between data on enhanced national TB surveillance systems and that held by the clinic and delays in obtaining data would recommend a closer liaison between clinical and public health services within the now established TB networks.

There are gaps in data monitoring. Sputum smear status is one of the most useful indicators of ongoing TB transmission. Mycobnet, the e-data source for the National Mycobacterial Reference Laboratories, collects data on culture and sensitivities of isolated strains of *Mycobacterium tuberculosis *but not on smear status. HIV testing for all those with TB has been recommended since 2000, but only the offer and not the result is recorded on the electronic databases and that only recently. However, in a larger data set, London contributed 57% of HIV co-infection predominantly in sub-Saharan Africans, born abroad and in their 30s [10]. Perhaps more importantly, testing for latent or active TB in patients with HIV infection is not yet employed, although a recent proposal for such testing has been made [10]. Outcome reporting has significantly improved compared to previous surveys [4]. Integration within the wider health services is especially important and, notably, the contribution of direct referral from radiology made a significant impact in Leicester, Birmingham and some London clinics.

The discrepancy between changes in incidence per 100, 000 and clinic TB numbers requires explanation. In most cities the higher increase in clinic numbers would be consistent with either improved case-finding or an underestimate of the population. The explanation for Bradford, where there has been an increase in incidence with almost stable numbers of TB cases requires that either the population is decreasing or patients are being treated outside the TB service, such as in primary care.

### Strain-typing

Increasing incidence could also be ascribed to the impact of micro-epidemics. From 1995-7 in London, clusters of cases accounted for 14.4% of culture-positive TB and were small (2-12 cases) [11]. An outbreak of isoniazid-resistant TB [12], the second largest in Europe and now numbering > 400 cases over a 15 year period with an identical strain [13], has highlighted the difficulty of containing TB in "hard-to-reach" groups [14]. However, typing and reporting to physicians occurred too late to make an impact on the outbreak, which followed its expected course [13,15,16]. Universal strain typing would have been especially useful in Manchester to determine whether the increase in infectious cases was due to recent transmission rather than reactivation or recent arrival in the UK. In Leicester, strain typing would have helped assess whether the increase in TB in the white ethnicity was due to infection with imported or local strains.

### Resources

Commissioning through primary care trusts (PCTs) has shown a good correlation between the figures relevant to their population and their resourcing of the local clinics. However, there was no significant correlation between fulfilment of targets in the national plan and the actual workload of the clinic. This discrepancy emphasises the need for the TB networks to be effective in sharing data and responding strategically across a city. Most cities reported that they had 1 nurse per 40 notifications, but four cities (Birmingham, Bradford, Manchester and Sheffield) with a previous or currently limited nursing service had seen increases in TB notifications (Table 5). Areas with fewer nurses showed the greatest increase in smear-positive cases (Figure 2). The provision of directly observed therapy (DOT) was patchy and loss to follow-up was significantly greater in those areas with fewer nurses. When limited, nursing time will focus on contact tracing. There will be less opportunity for DOT, assistance with complex needs, educational outreach and screening new entrants. Although the Cochrane reviews have not found good evidence to support DOT [6], the constant contact between TB nurses provides not just reassurance that patients are taking medication, but also social and health care support, especially for those with complex needs and associated morbidities. These are the first data to confirm that the target of 1 nurse per 40 notifications does have a significant effect on TB control. Multi-drug resistance (MDR) has a significant effect on resource allocation, but costs are largely in terms of inpatient stay and drugs with smaller increases in costs for DOT due to the duration of treatment. However, in the UK, costs for MDRTB should be allocated through a national scheme of specialist commissioning in order not to detract from standard TB services, although this is not the case in Manchester and Leeds.

### International comparisons

Four aspects of a TB control programme in large cities were identified in Barcelona [17]. Epidemiological surveillance across a city has been an important first step, along with assessment of outcome of treatment. Contact tracing is an essential part of the TB control programme in the UK, but is currently not well documented. Plans to have an electronic record of contact tracing linked with strain typing are currently ongoing. Social support is readily accessible for residents in the UK, but only certain areas have been able to negotiate support for illegal immigrants on the grounds that active TB in this group represents a risk to the nation's public health. In assessing the TB health programme in UK cities against international standards set by the World Health Organization [18], service delivery and workforce in terms of access and coverage of TB patients have been highlighted as problems leading to a reduced responsiveness to the increase in tuberculosis. Similar problems were noted in outbreaks amongst the homeless of Paris [19]. Variations in TB control programmes have been noted in Western European cities, with regard to new entrant screening, use of DOT and outcome recording, although no link was made with available resources [20]. In their most recent annual report [21], New York City statistics have shown a steep decline in TB cases since 1992 due to investment and implementation of policies to improve case-finding, case-holding, a legal framework to ensure adherence and collaboration with social organizations offering support. Notification of TB suspects and targets set for the number of contacts to be examined per index have not been commonly adopted in Western European cities compared to other aspects of their integrated TB control programme. Adequate provision of resources in New York City, in excess of $1 billion, has been the most notable difference compared to most large cities [22].

### Limitations

London is a complex set of communities and the number of PCTs with > 100 cases per annum exceeds that for the rest of the UK; further detailed analysis is recommended.

## Conclusions

Local epidemiology is important and requires sufficient clinical detail to inform TB control strategies. A ratio of 40 notifications or less per TB nurse must be implemented nationally (the current TB control documents do not specify this target [5,6]. Only then can realistic improvements in case detection and case-holding be effective.

## List of abbreviations

CCDC: consultant in communicable disease control; CIEC: consultant in infection and environmental control; DNA: deoxyribonucleic acid; DOT: directly observed therapy; ETS: enhanced TB surveillance; HIV: human immunodeficiency virus; HPA: Health Protection Agency (public health); MDT: multidisciplinary team; NC: north central; NE: north-east; NICE: National Institute for Health and Clinical Excellence; PA: professional activity (4 h of time assigned to a role in a job description); PCT: Primary Care Trust (commissioners of health care); TB: tuberculosis; UK: United Kingdom.

## Competing interests

The authors declare that they have no competing interests.

## Authors' contributions

GB had full access to the data and is responsible for the data analysis. Original study concept: GB. Study design: GB & MK. Acquisition of data: all authors. Analysis and interpretation of data: GB and MK overall; all authors for data relating to their cities

Statistical analysis: GB. Administrative, technical and material support: GB.

All authors have read and approved the final manuscript.

## Pre-publication history

The pre-publication history for this paper can be accessed here:

http://www.biomedcentral.com/1471-2458/11/896/prepub
